# SETD3 acts as a prognostic marker in breast cancer patients and modulates the viability and invasion of breast cancer cells

**DOI:** 10.1038/s41598-020-59057-5

**Published:** 2020-02-10

**Authors:** Nourhan Hassan, Niklas Rutsch, Balázs Győrffy, Nancy Adriana Espinoza-Sánchez, Martin Götte

**Affiliations:** 1 Department of Gynecology and Obstetrics, Münster University Hospital, Albert-Schweitzer-Campus 1, D11, Münster, 48149 Germany; 2 Department of Bioinformatics and 2nd Department of Pediatrics, Semmelweis University, and TTK Momentum Cancer Biomarker Research Group, Budapest, Hungary; 3 Unidad de Investigación en Virología y Cáncer, Hospital Infantil de México Federico Gómez, Ciudad de México, 06720 Mexico

**Keywords:** Mechanisms of disease, Prognostic markers

## Abstract

In several carcinomas, the SET Domain Containing 3, Actin Histidine Methyltransferase (SETD3) is associated with oncogenesis. However, there is little knowledge about the role of SETD3 in the progression and prognosis of breast cancer. In this study, we first analyzed the prognostic value of SETD3 in breast cancer patients using the database of the public Kaplan-Meier plotter. Moreover, *in vitro* assays were performed to assess the role of SETD3 in the viability and capacity of invasion of human breast cancer cell lines. We observed that the high expression of SETD3 was associated with better relapse-free survival (RFS) of the whole collective of 3,951 patients, of Estrogen Receptor-positive, and of Luminal A-type breast cancer patients. However, in patients lacking expression of estrogen-, progesterone- and HER2-receptor, and those affected by a p53-mutation, SETD3 was associated with poor RFS. *In vitro* analysis showed that SETD3 siRNA depletion affects the viability of triple-negative cells as well as the cytoskeletal function and capacity of invasion of highly invasive MDA-MB-231 cells. Interestingly, SETD3 regulates the expression of other genes associated with cancer such as β-actin, FOXM1, FBXW7, Fascin, eNOS, and MMP-2. Our study suggests that SETD3 expression can act as a subtype-specific biomarker for breast cancer progression and prognosis.

## Introduction

Breast cancer has a large global impact, accounting for 15% of cancer deaths in women worldwide^1^. Although newly introduced molecularly targeted therapies such as PARP, PI3K/AKT/mTOR and other kinase inhibitors as well as immunotherapy allow for better treatment of breast cancer, much remains to be understood^2^. Not only through the introduction of more specific morphologic subtypes, but also through a thorough molecular classification, breast cancer therapy has evolved towards targeted therapies with a more favorable side-effects profile^3,4^. This advance is largely exemplified by biomarkers such as estrogen receptor (ER) or human epidermal growth factor receptor 2 (HER2), which serve as decision support tools. Indeed, these biomarkers are excellent examples of functionally relevant prognostic markers that have been successfully utilized as therapeutic targets in breast cancer^5,6^.

Among potentially relevant novel biomarkers that can expand the diagnostic repertoire in malignant disease, factors modulating the cytoskeleton are prime candidates, as they may have a direct impact on cell motility and metastasis, which is the leading cause of death in cancer patients^7–9^. Novel evidence has highlighted the relevance for posttranslational methylation for cytoskeletal function^10^. Post-translational modifications such as methylation modulate the function of numerous eukaryotic proteins^11^. Most information in this field refers to the methylation of histones on arginine and lysine residues and helped to better understand the epigenetic control of gene expression^12^. However, it has been long known that non-histone proteins like beta-actin are methylated as well^13^. Methylation may, therefore, constitute a regulatory mechanism that does not only take place at the level of DNA expression. Recently, the SET Domain Containing 3, Actin Histidine Methyltransferase (SETD3) has been identified to be an actin-specific histidine N-methyltransferase^10,14^. SETD3 is a ubiquitously expressed protein, which is able to methylate the His73 position of actin in a physiologically relevant manner by using the co-factor S-adenosyl-methionine (SAM) as a methyl group donor^10^. Structural analysis has shown that actin methylation regulates the flexibility and stability of the interdomain of actin^15^. SETD3 does not affect the basal function of actin, as a knockout mouse model (SETD3^−/−^) is still viable. However, homozygous knockout females showed primary dystocia. These findings suggest that the phenotype of irregular uterine contractions may be caused by the lack of actin methylation^10^. Importantly, altered regulation of actin is an important step during pre-malignant stages, as actin is one of the key proteins driving cancer cell migration^16,17^. In order for cells to invade and metastasize they need to acquire the capacity to migrate^18^. Migratory single cells can show amoeboid or mesenchymal movement, whereas groups of cells can move in the form of collective migration. In both cases, the re-organization of actin is a critical step.^19^.

Besides regulating actin, SETD3 shows various additional protein interactions, while its role in cell signaling remains unclear. In breast cancer, the forkhead box protein M1 (FoxM1) has tumor-promoting functions impacting patient prognosis^20^. FoxM1 is also methylated by SETD3, claiming a core position in oncogenesis^21^. It was demonstrated that SETD3 is a factor with relevance to cell cycle regulation. It has an expression peak in the S-phase and underlies a regulatory degradation by F-box/WD repeat-containing protein 7 beta isoform (FBXM7-beta) and glycogen synthase kinase 3 beta isoform (GSK3-beta). Inhibition or depletion of these two factors results in elevated SETD3 levels and promotes cell proliferation. This concurs with elevated SETD3 levels which have been found in human liver cancer^22^. Supporting findings suggest that SETD3 is required for p53 target recruitment and activation^23^. This identifies several relevant targets; the phenotypical and physical properties due to changes in beta-actin methylation (yet characterized by primary dystocia) and signaling properties of SETD3 that may affect oncogenesis^10,21^. Based on this evidence, we hypothesize that alterations in SETD3 expression may affect cytoskeletal function, cancer cell motility and clinical outcome in breast cancer patients. As there are multiple regulatory mechanisms in which SETD3 is involved, it is expected to define specific subgroups that exhibit a strong effect of SETD3 in terms of prognosis.

In this study, we first used the Kaplan Meier Plotter resource^24^ incorporating the gene expression data of over five thousand breast cancer tumor samples, to correlate SETD3 expression to the survival of breast patients stratified by different clinical subtypes. Then, we employed SETD3 siRNA knockdown *in vitro* in a range of well-established human mammary carcinoma cell lines to analyze the role of SETD3 depletion in cytoskeleton-associated functions with relevance to malignant cell behavior. Our data assign a prognostic value to altered SETD3 expression in breast cancer that depends on p53 mutation status and provides proof for a role of SETD3 in the viability, motility, cytoskeletal organization, and invasiveness of triple-negative breast cancer cells.

## Results

### Patients with triple-negative and p53 mutated tumors expressing high levels of SETD3 have a poor outcome

**Figure 1:**
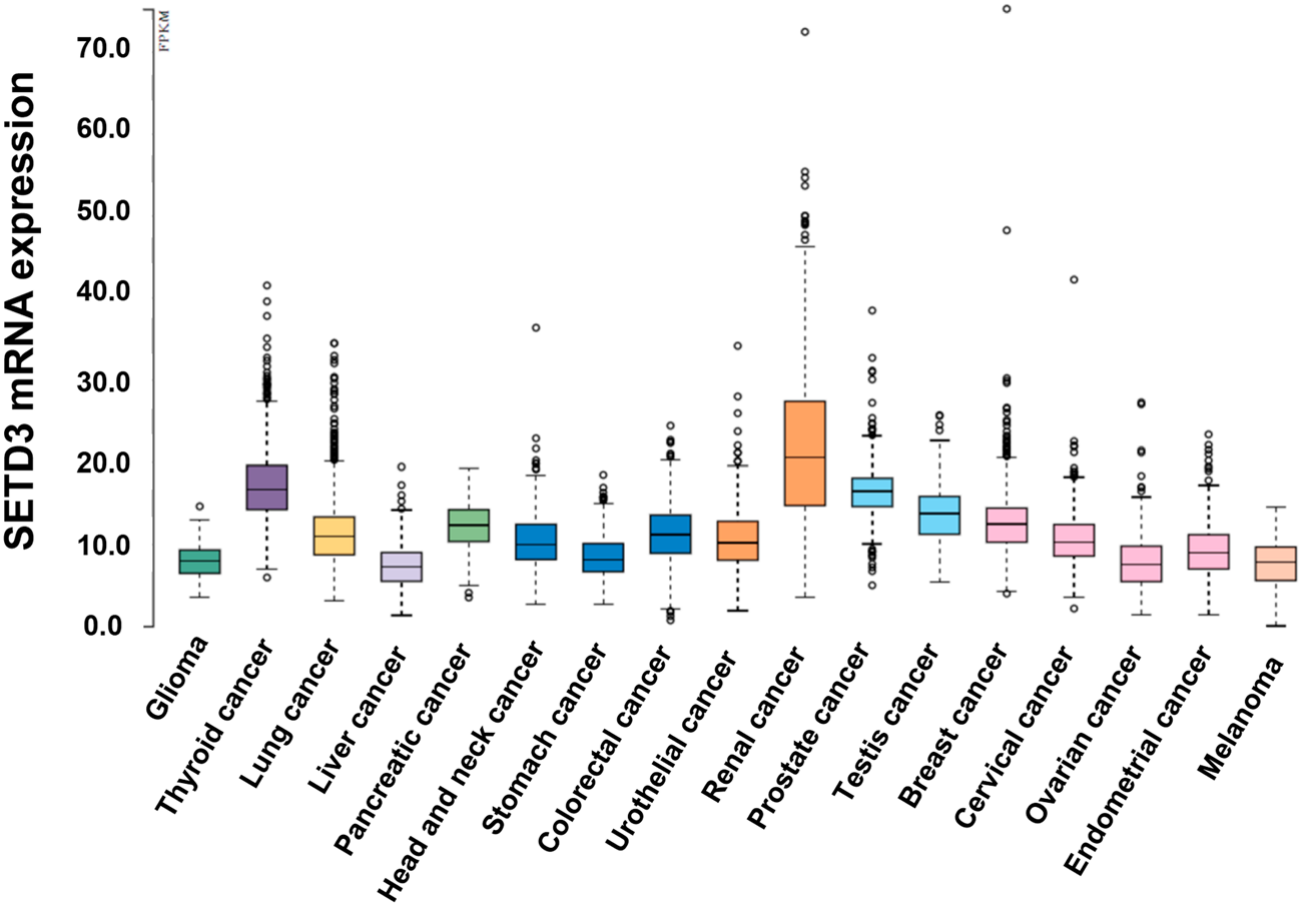
TCGA analysis of SETD3 expression in different types of cancers. Frequency of SETD3 expression in different types of solid tumors. RNA-seq data in 17 cancer types are plotted as median number fragments per kilobase of exon per million reads generated by The Cancer Genome Atlas (TCGA). Points are displayed as outliers if they are above or below 1.5 times the interquartile range. The cancer types are color-coded according to which type of normal organ cancer originates from.

To have an overview of SETD3 expression in different solid tumors, we analyzed its expression in samples from the TCGA, which demonstrated that SETD3 is mainly expressed in renal and thyroid cancer (Fig. 1). However, in cancers associated with women, we observed higher expression of SETD3 in breast cancer compared to cervical, endometrial and ovarian cancer (Fig. 1).

**Figure 2:**
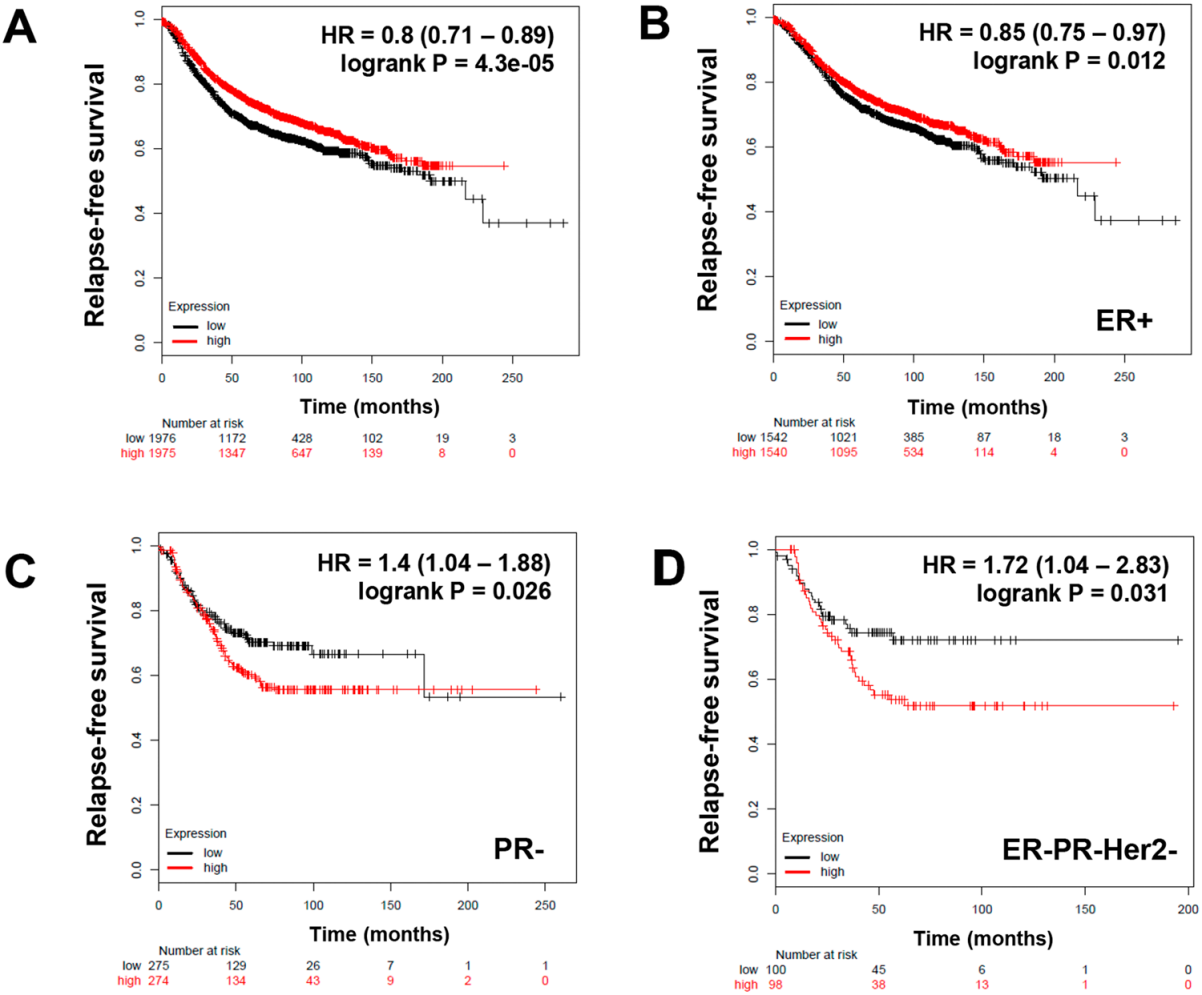
The prognostic value of the expression of SETD3 in patients with breast cancer stratified by hormone receptor status. Kaplan-Meier relapse-free survival curves are plotted based on: (**A**) all breast cancer patients (n = 3951), (**B**) Estrogen receptor (ER) positive status, ER+ (n = 3,082) (**C**) Progesterone receptor negative (PR) status, PR- (n = 549), and (**D**) triple-negative expression of ER, PR, and Her2 (n = 196). Log-rank p values and hazard ratios (HRs; 95% confidence interval in parentheses) are shown. The corresponding Affymetrix IDs is: 212465_at_SETD3.

In order to establish whether SETD3 expression could have an impact on breast cancer prognosis, we investigated its relation to relapse-free survival (RFS). Gene expression data from tumor samples of 3,951 patients without any stratification and patients classified according to different molecular subtypes and receptor status (Table 1) were analyzed using the online tool Kaplan Meier Plotter^24^. Table 1 shows the HR (Hazard ratio) and *P* values of SETD3 in each classification. We observed that over all samples, high SETD3 levels were a protective factor for patient survival with an HR = 0.8; *P* = 4.3e-05 (Fig. 2A). This applied to ER-positive tumors only (HR = 0.85; *P* = 0.012) (Fig. 2B) and to the intrinsic subtype Luminal A (HR = 0.75; *P* = 0.0049) (Table 1). However, in patients with PR-negative tumors, high SETD3 expression correlated with a worse survival (HR = 1.4; *P* = 0.026) (Fig. 2C), while in the other groups associated with hormone receptor or Her2 expression, we did not find a correlation (Table 1). Of note, in the clinically highly relevant subgroup of triple-negative tumors, the expression of SETD3 was associated with poor relapse-free survival (HR = 1.72; *P* = 0.031) (Fig. 2D).

**Table 1:** Correlation between the expression of SETD3 and the status of breast cancer patients according to different classifications.

Classification	status	Cases	HR 95% CI	*P* value
ALL		3951	0.8 (0.71–0.89)	**4.3e-05**
Estrogen receptor (ER)	Positive (+)	3082	0.85 (0.75–0.97)	**0.012**
	Negative (−)	869	1.03 (0.83–1.27)	0.79
Progesterone receptor (PR)	Positive	589	1.29 (0.9–1.82)	0.16
	Negative	549	1.4 (1.04–1.88)	**0.026**
Her2	Positive	252	1.15 (0.75–1.78)	0.52
	Negative	800	1.15 (0.88–1.49)	0.31
ER, PR, Her2	Negative	196	1.72 (1.04–2.83)	**0.031**
Intrinsic subtype	Luminal A	1933	0.75 (0.66–0.93)	**0.0049**
	Luminal B	1149	1.01 (0.84–1.23)	0.89
	Her2	251	1.1 (0.75–1.62)	0.62
	Basal	618	1.01 (0.79–1.3)	0.91
Lymph node	Positive	1133	1.04 (0.86–1.27)	0.68
	Negative	2020	0.97 (0.82–1.15)	0.74
Grade	1	345	0.81 (0.48–1.36)	0.42
	2	901	0.98 (0.77–1.25)	0.87
	3	903	1.11 (0.89–1.37)	0.37
p53	Mutated	188	2.1 (1.29–3.44)	**0.0024**
	Wild type	273	0.95 (0.63–1.45)	0.82
p53 mutated	ER+	87	2.93 (1.47–5.85)	**0.0014**
	ER-	101	1.46 (0.72–2.93)	0.29
	PR+	25	11.23 (1.42–88.83)	**0.0039**
	PR-	50	2.15 (0.86–5.4)	0.95
	Luminal A	164	2.35 (0.8–6.88)	0.11
	Luminal B	55	2.42 (1.01–5.79)	**0.041**
p53 wild type	ER+	251	0.94 (0.61–1.46)	0.8
	ER-	22	1.88 (0.34–10.37)	0.46
	PR+	52	3.18 (0.87–11.65)	0.065
	PR-	24	0.77 (0.2–2.87)	0.69
	Luminal A	32	0.89 (0.5–1.59)	0.69
	Luminal B	87	0.91 (0.47–1.76)	0.77

We next determined the correlation of SETD3 expression with nodal status (Table 1). We found that the HR of the expression of SETD3 had a high score but that it was not significantly related to the survival of the patients (Table 1). The treatment decision and the prognosis of a breast cancer patient depend on many factors including the grade of the tumor associated with LN status^25^. Like in our analysis regarding nodal status, we did not find a correlation between SETD3 expression and the grade of the tumor (Table 1).

**Figure 3:**
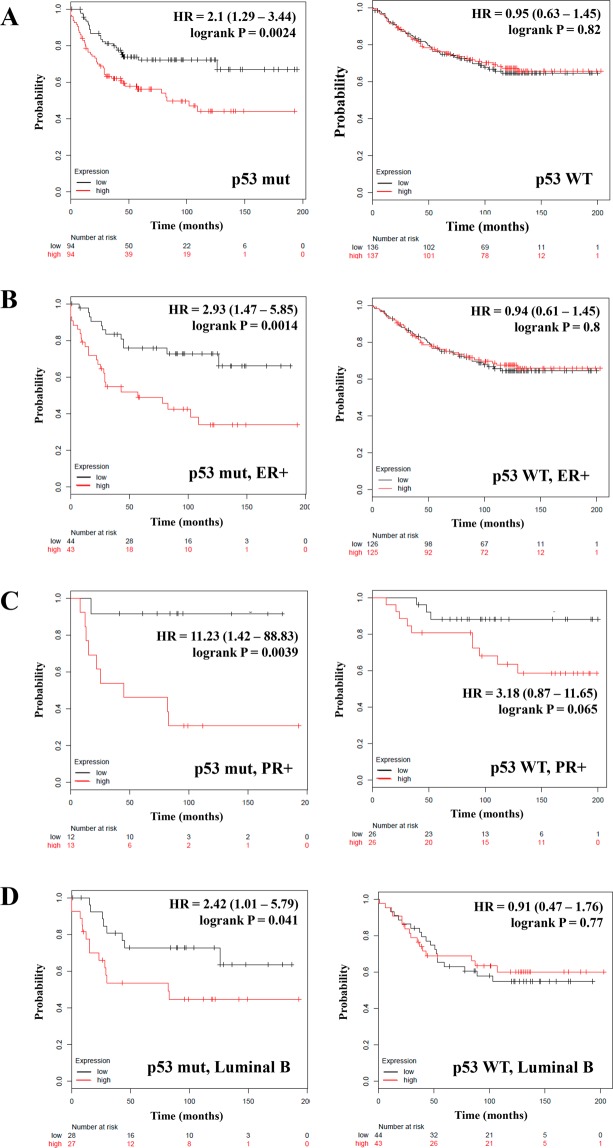
The prognostic value of the expression of SETD3 in patients with breast cancer stratified by p53 status. Kaplan-Meier relapse-free survival curves are plotted based on (**A**) p53 status, p53 mutated (n = 188) and according to the status of p53 and the expression of (**B**) ER, (**C**) PR and (**D**) the intrinsic molecular classification Luminal B. Log-rank p values and hazard ratios (HRs; 95% confidence interval in parentheses) are shown. The corresponding Affymetrix IDs is: 212465_at_SETD3.

Since SETD3 is a positive regulator of p53^23^, we studied its expression in our patient collective stratified by p53 status. In patients with tumors in which p53 is mutated, the high expression of SETD3 correlated with inferior RFS (HR = 2.1; *P* = 0.0024) (Fig. 3A, left panel), but we did not find a correlation in the wild type (WT) p53 group (HR = 0.95; *P* = 0.82) **(**Fig. 3A, right panel and Table 1**)**.Taking a closer look at the expression of SETD3 and p53 in patients with ER-, and PR-positive or negative tumors and the Luminal A and B classification, we observed that in patients with ER- and PR-positive and p53 mutated tumors, a high expression of SETD3 was associated with worse RFS (HR = 2.93; *P* = 0.0014, and HR = 11.23; *P* = 0.0039, respectively) (Fig. 3B,C, left panel). Also, in p53 mutated Luminal B tumors, but not in Luminal A tumors, SETD3 correlated with shorter survival (HR = 2.42; *P* = 0.041) (Fig. 3D, left panel and Table 1). Again, we did not find any correlation of SETD3 with survival in p53 WT and ER-, PR-negative and Luminal A or Luminal B tumors (Fig. 3B–D, right panel and Table 1). Our analysis results suggest that the expression of SETD3 influences the clinical outcome according to the expression of hormone receptors and p53 status. We can also conclude that the function of SETD3 depends on the presence of mutations and the expression patterns of other genes in breast cancer.

### Expression of SETD3 in cell lines representing different molecular classifications of breast cancer

**Figure 4:**
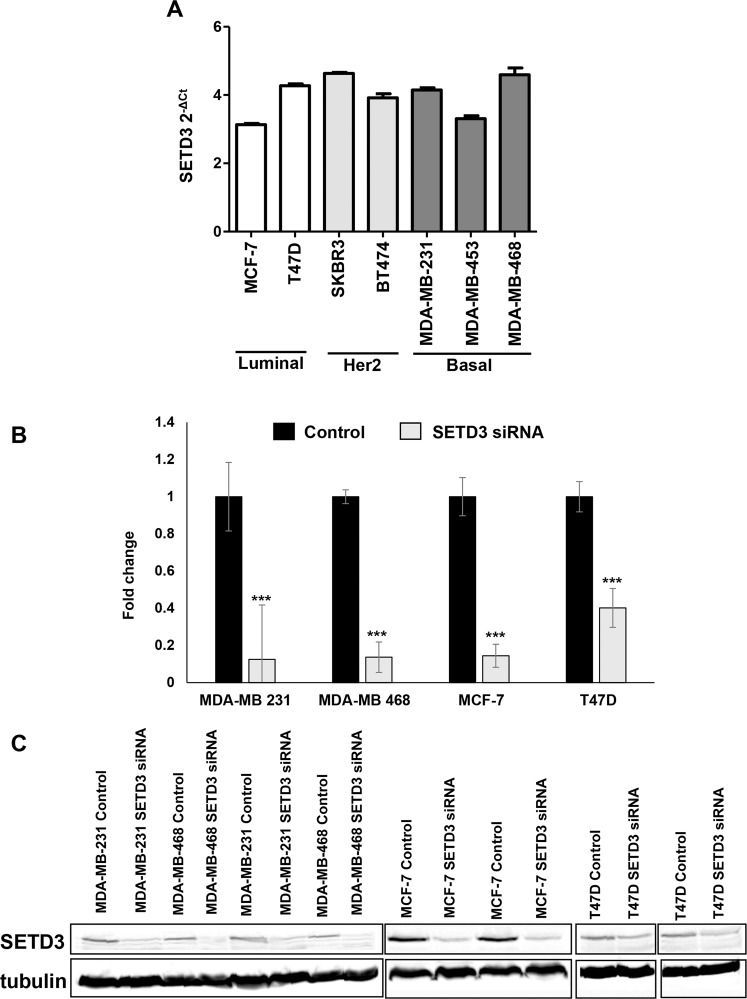
Expression of SETD3 in breast cancer cell lines. (**A**) Relative expression of *SETD3* was quantified by qRT-PCR in 7 breast cancer cell lines representative of the luminal (MCF-7 and T47D), Her2-positive (BT474 and SKBR3), and basal (MDA-MB-231, -453 and -468) subtype. Individual experiments were normalized against *β-ACTIN* and the relative expression was represented by 2-ΔCt. (**B**,**C**) The expression of SETD3 was blocked with an siRNA in basal breast cancer cell lines MDA-MB-231 and -468, and Luminal cell lines MCF-7 and T47D. Knockdown of SETD3 was confirmed by B) qRT-PCR and C) western blot. Western blot images are composites of individual blots as indicated by separated boxes. Tubulin signals are derived from the same stripped and reprobed membranes as the corresponding SETD3 signals shown in the upper panels. Full-length blots are shown in the Supplementary File, Supplementary Fig. S1. Data represent the mean ± SEM (standard error of the mean) from 3 independent experiments in triplicates. Bars with asterisks represent comparisons with statistically significant differences (*P* < 0.05).

As a prerequisite for studying SETD3 function *in vitro*, we characterized its expression by quantitative real-time PCR (qPCR) in a panel of seven representative human mammary cancer cell lines. The model cell lines were classified as follows: *Luminal*, T47D and MCF-7; *Her2 enriched*, SK-BR-3 and BT-474, and *Basal*, MDA-MB-231, MDA-MB-468 and MDA-MB-453^26^. Only MCF-7 cells showed a p53 WT status. SETD3 was expressed in all cells and no significant differences in the relative expression (represented by 2^-ΔCt^) were noted between the subtypes and within each subtype (Fig. 4A). Taking these results into account, we transfected only the Luminal cells T47D and MCF-7, and the basal cells MDA-MB-468 and MDA-MB-231 with a small interfering RNA (siRNA) to silence the expression of SETD3. Forty-eight hours after transfection, we observed that all cell lines downregulated the expression of SETD3 compared to control, almost 0.6 fold in T47D and 0.9 fold in the other cell lines (Fig. 4B). By performing western blot, we confirmed that in all cells SETD3 siRNA substantially decreased the levels of SETD3 protein (Fig. 4C), and was therefore found suitable to study the functional consequences of SETD3 depletion.

### SETD3 differentially affects viability and invasiveness of mammary carcinoma cells in a subtype-specific manner

**Figure 5:**
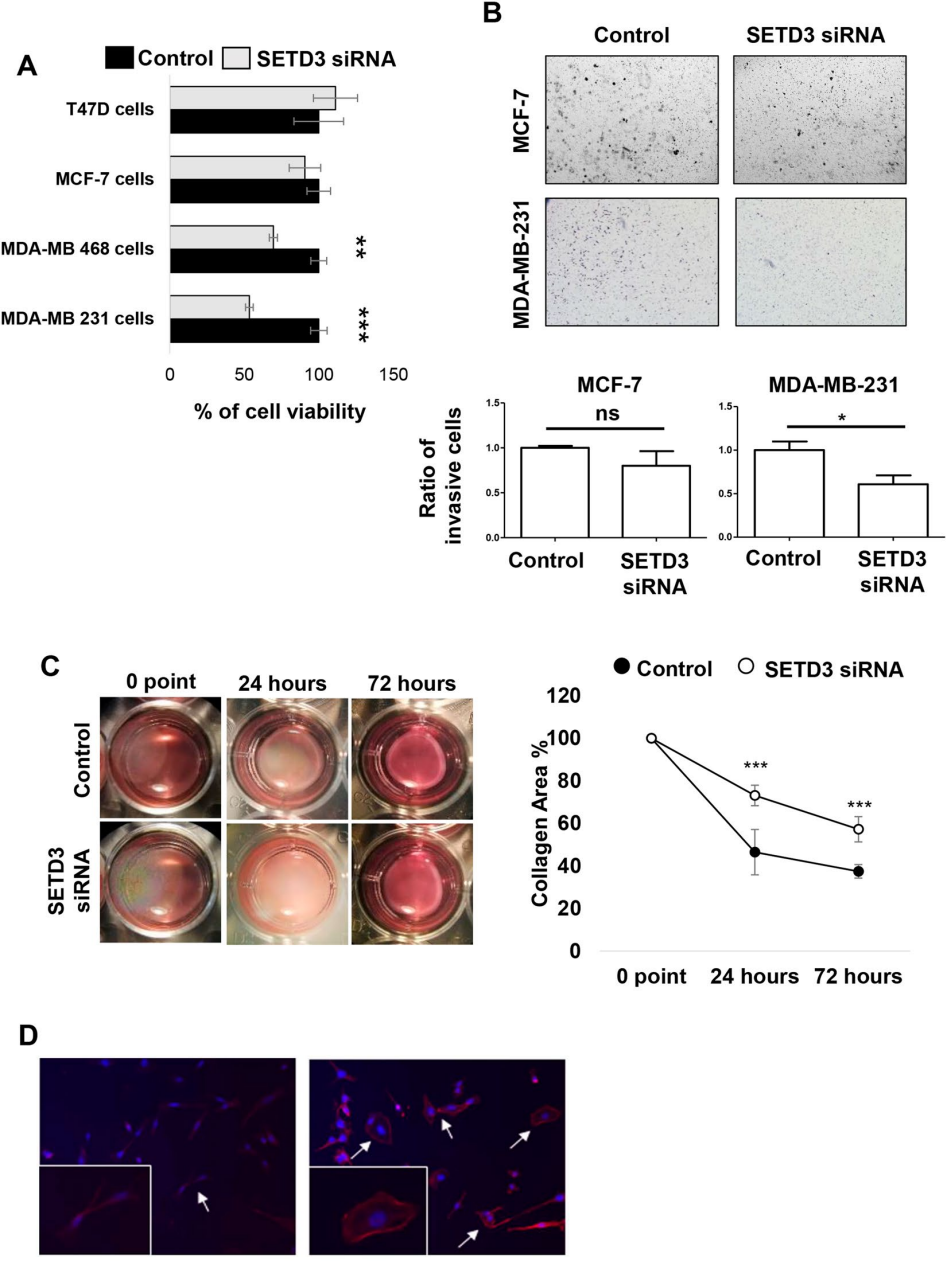
The inhibition of SETD3 expression blocks the viability and invasive phenotype of MDA-MB-231 breast cancer cells. (**A**) MTT assay reveals a significant effect of SETD3 on cell viability in the aggressive basal cell lines MDA-MB-231 and MDA-MB-468. (**B**) After 48 hours of culture with siRNA to inhibit the expression of SETD3, a Matrigel transwell invasion was performed. MCF-7 cells are poorly invasive luminal cells while MDA-MB-231 cells are highly invasive basal cells. (**C**) Collagen contraction, and (**D**) immunofluorescence assays were performed in MDA-MB-231 cells. Upper (**B**) and left (**C**) panels show representative images of invasion and collagen contraction respectively, and the  ratio of invasive cells or the percent of contraction collagen area were graphed. Data represent the mean ± SEM from 3 independent experiments in triplicates. Bars or points with asterisks represent comparisons with statistically significant differences (*P* < 0.05). For immunofluorescence (**D**) representative images are shown. Actin filaments were stained with phalloidin (red) and nuclei with 4′,6′-diamidino-2-phenylindole (DAPI, blue). White arrows represent the normal cells (left panel) and those that have modified their morphology after the depletion of SETD3 (right panel). Magnification 200×.

After the initial characterisation of our experimental system, we aimed to uncover the possible participation of SETD3 in processes associated with tumor progression such as proliferation and invasion^27^. Following the downregulation of SETD3 expression, MTT assays revealed that the basal-type cell lines had the lowest viability: MDA-MB-231 showed a significant reduction by 50% compared to control siRNA-treated cells, and MDA-MB-468 displayed a reduction of viability by 35%. (Fig. 5A). In contrast, cell viability was not significantly altered in the Luminal cell lines (Fig. 5A). MDA-MB-231 is a highly aggressive breast cancer cell line according to its invasive and metastatic properties while MCF-7 is not^28^. Due to these properties, we used them as a model to study the role of SETD3 in invasion using the established Matrigel invasion chamber assay. As shown in Fig. 5B, SETD3 siRNA treated MDA-MB-231 cells reduced their invasive potential by 40% with respect to control. In contrast, SETD3 siRNA did not significantly reduce the invasion characteristics of MCF-7 cells. Because the reduction in SETD3 expression affected the invasion capacity only in MDA-MB-231 cells, we performed a collagen contraction assay in this cell line. The capacity to contract collagen I gels was diminished by SETD3 siRNA-treatment compared to control after 24 and 72 hrs in the triple-negative cell line (Fig. 5C), indicating a possible defect in cytoskeletal function. As SETD3 is involved in cytoskeletal rearrangement^14^, which is implicated in migration and invasion^17^, we performed immunofluorescence microscopy employing the fluorescently labeled actin-binding protein phalloidin to study the consequence of SETD3 knockdown on cell morphology. In agreement with the invasion and collagen contraction assays, both cell morphology and the structure of actin filaments were altered when SETD3 was knocked down (Fig. 5D). While the control cells had an elongated, mesenchymal-like morphology typical of the invasive cell line, a proportion of the SETD3-depleted cells showed a round morphology (Fig. 5D). Overall, these results demonstrate a role for SETD3 in cytoskeletal organization and function of aggressive breast cancer cells, which correlates with our observation that the expression of SETD3 in triple negative tumors was related to poor prognosis (Fig. 2D).

### SETD3 regulates the expression of different effector genes with relevance to cancer progression

**Figure 6:**
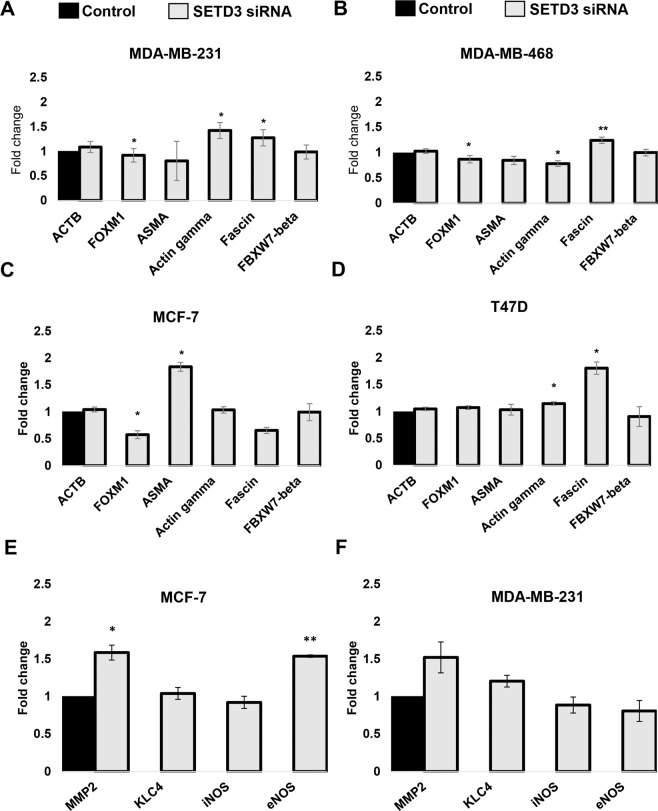
SETD3 regulates the expression of different potential effector genes. SETD3 siRNA was transfected into MDA-MB-231 (**A**), MDA-MB-468 (**B**), MCF-7 (**C**) and T47D (**D**) cells and the expression of *ACTB* (*β-actin*), *FOXM1, ASMA, Gamma-actin, Fascin*, and *FBXW7-beta* was analyzed. The expression of *MMP-2, KLC4, iNOS, and eNOS* were analyzed in MCF-7 (**E**) and MDA-MB-231 (**F**). Individual experiments were normalized against *GAPDH* and the fold change was performed using the ∆∆Ct method taking the SETD3 expressed cells as a control. Data represent the mean ± SEM from 3 independent experiments in duplicates. Bars with asterisks represent comparisons with statistically significant differences (*P* < 0.05).

It is known that SETD3 regulates the expression of genes related to normal physiology, development and cancer progression such as *FOXM1* (Forkhead Box M1), *ACTB* (β-actin), *ASMA* (alpha-smooth muscle actin), *ACTG* (λ-actin), *FSCN* (Fascin Actin-Bundling Protein), *FBXW7* (F-Box and WD Repeat Domain Containing 7)^10,22,29–32^ but it is not yet known whether this regulation also occurs in breast cancer. To find out if SETD3 influences the expression of the genes listed above in our *in vitro* system, we transfected SETD3 siRNA into the basal cells MDA-MB-231 and MDA-MB-468, and Luminal MCF-7 and T47D cells. Our results show that in all SETD3 siRNA treated cell lines, some genes are significantly up- or down-regulated, however, this regulation occurred in a cell-type-specific manner, and was quantitatively modest in most cases (Fig. 6). In MDA-MB-231 cells, Gamma-actin was up-regulated 0.4 fold, while Fascin was 0.2 fold up-regulated, and FOXM1 was downregulated 0.1 fold (Fig. 6A). In MDA-MB-468 cells FOXM1 and Gamma-actin were downregulated 0.1 and 0.2 fold respectively while Fascin was up-regulated 0.3 fold (Fig. 6B). With respect to Luminal cells, MCF-7 FOXM1 was downregulated 0.5 fold and ASMA was upregulated 0.84 fold (Fig. 6C), and in T47D cells Fascin was upregulated 0.9 fold, whereas Gamma-actin was up-regulated 0.1 fold (Fig. 6D). Also, to analyze the gene expression of additional molecules regulated by SETD3^10,22,29–32^ that could potentially explain the differences between the prognostic impact on diverse breast cancer subtypes, we also quantified MMP-2, KLC4, iNOS and eNOS mRNA levels in MCF-7 and MDA-MB-231 cells. We found that MMP-2 was up-regulated in the absence of SETD3 in both cell lines, however, this regulation was only significant in the luminal MCF-7 cell line (Fig. 6E,F). In addition, eNOS was up-regulated only in MCF-7 (Fig. 6E). According to our data, we can suggest that FOXM1 is the most homogeneously regulated gene affected by SETD3 regardless of the aggressiveness or classification of the cell lines and that numerous genes are affected by SETD3 knockdown in a cell-type-specific manner.

### Functional enrichment analysis for SETD3 related proteins

**Figure 7:**
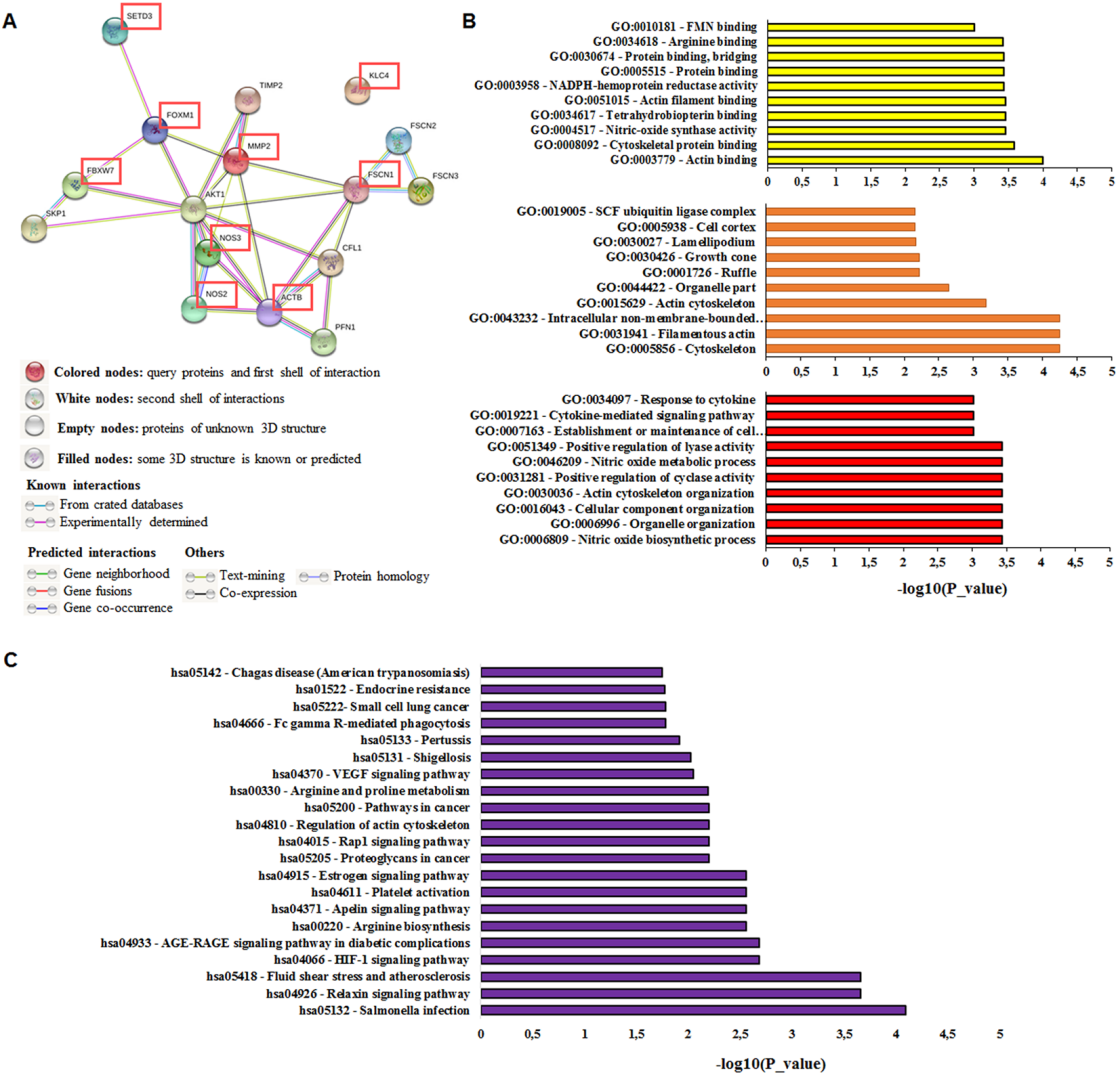
The network of SETD3, FOXM1, FBXW7, ACTB (β-actin), Fascin, MMP-2, KLC4, iNOS, and eNOS interactors. **(A**) STRING database output depicting functional and physical interactors of SETD3, FOXM1, FBXW7, ACTB, and Fascin obtained from http://string-db.org/. The analyzed proteins are highlighted in red boxes. (**B**) GO (gene ontology) analysis of SETD3, FOXM1, FBXW7, ACTB (β-actin), Fascin, MMP-2, KLC4, iNOS, and eNOS. The 10 most significantly (p < 0.05) enriched GO terms in molecular function (yellow), cellular component (orange), and biological process (red) branches are presented. (**C**) KEGG pathway analysis. All the adjusted statistically significant values of the terms were negative 10-base log-transformed.

To find functional interaction networks of SETD3 and its potential effector genes such as FOXM1, FBXW7, ACTB (β-actin), Fascin, MMP-2, KLC4, eNOS, and iNOS, we used the online bioinformatic tool STRING^33^. The STRING analysis showed that SETD3 has close interactions only with FOXM1, while ACTB has interactions mainly with PFN1 and CFL1, but also has close interactions with Fascin 1–3 and is interconnected with NOS2 (iNOS) and NOS3 (eNOS). The rest of the molecules are also interconnected. FOXM1 is highly interconnected with FBXW7, AKT1, and MMP2. On the other hand, FBXW7 is associated with SKP1 and AKT1. Finally, KLC4 is not related to any of the molecules (Fig. 7A). Figure 7B and Table S1 show the molecular function, the cellular component and the biological process of the analyzed proteins. Interestingly, we identified enriched pathways linked to cancer such as VEGF, proteoglycans, Rap1, estrogen, and HIF-1, parasite and bacterial infection, in other pathological processes such as diabetes and atherosclerosis, and normal processes including arginine biosynthesis (Fig. 7C and Table S2). Table S3 shows the 10 most significantly enriched terms based on PubMed co-citation analysis^33^, which also includes several terms linked to cancer.

### The expression of FOXM1 has a negative impact on the survival of breast cancer patients and does not depend on nodal status and tumor grade

SETD3 regulates the expression of and has very close interactions with FOXM1 according to our STRING analysis, and simultaneous expression of SETD3 and FOXM1 was linked to tumorigenicity in an *in vivo* model of liver cancer^22^. Our data indicate that the expression of FOXM1 negatively influences the relapse-free survival of the whole study collective (HR = 1.67; *P* = <1E-16) as well as in ER-positive (HR = 1.81; *P* = <1E-16) (Supplementary Fig. 2A, left panel), PR-positive (HR = 2.66; *P* = 1.3e-07) and Her2-negative tumors (HR = 1.81; *P* = 1.1e-05) (Table 2). When we analyzed the expression of *FOXM1* in patients based on the intrinsic subtypes Luminal A and B, we observed a poor relapse-free survival with an HR = 1.81; *P* = 8.5e-13 in the Luminal A group (Supplementary Fig. 2A, right panel) and a HR = 1.36; *P* = 0.0017 in the Luminal B group (Table 2). Interestingly, the expression of *FOXM1* correlated with reduced RFS of breast cancer patients regardless of their nodal status and tumor grade (except for Grade III) (LN-positive: HR = 1.74, *P* = 3.9e-08; LN-negative: HR = 1.7, *P* = 9.1e-10; Grade I: HR = 3.31, *P* = 3.7e-05; Grade II: HR = 1.55, *P* = 0.00034) (Table 2). In contrast, in patients with estrogen receptor-negative tumors and in the molecular subtype Her2, higher *FOXM1* was associated with better survival (HR = 0.75; *P* = 0.0087; HR = 0.63; *P* = 0.021, respectively). Although the correlation of this gene with p53 mutated tumors was negative, in p53 WT cancers, high *FOXM1* expression correlated with worse RFS (HR = 1.66; *P* = 0.019) (Table 2). Taking into account that SETD3 regulates the expression of FOXM1, we analyzed the co-expression of SETD3/FOXM1 only in ER-positive and Luminal A tumors where SETD3 expression correlated with better RFS (Fig. 2B), with the purpose of investigating if the interaction of both genes affects the survival of patients. We observed that the co-expression of both genes correlates with worse survival in ER-positive tumors (HR = 1.22; *P* = 0.028) while in the Luminal A subgroup, we did not find a significant correlation (HR = 1.02; *P* = 0.84) (Supplementary Fig. 2B, left and right panel). We conclude that although we observe that SETD3 regulates FOXM1 in cell lines regardless of their classification, the expression of FOXM1 in tumors is sufficient to confer worse patient survival even when SETD3 (which is associated with better survival) is co-expressed. We suggest that within a tumor the regulation of FOXM1 may not only be carried out by SETD3 but also through other molecules.

**Table 2:** Correlation between the expression of FOXM1 and the status of breast cancer patients according to different classifications.

Classification	status	Cases	HR 95% CI	*P* value
ALL	—	3,951	1.67 (1.5–1.87)	**<1E-16**
Estrogen receptor	Positive	3,082	1.81 (1.81–2.06)	**<1E-16**
	Negative	869	0.75 (0.61–0.93)	**0.0087**
Progesterone receptor	Positive	589	2.66 (1.82–3.87)	**1.3e-07**
	Negative	549	1.2 (0.9–1.6)	0.22
Her2	Positive	252	0.77 (0.49–1.18)	0.23
	Negative	800	1.81 (1.38–2.36)	**1.1e-05**
Intrinsic subtype	Luminal A	1,933	1.88 (1.58–2.25)	**8.5e-13**
	Luminal B	1,149	1.36 (1.12–1.65)	**0.0017**
	Her2	251	0.63 (0.43–0.94)	**0.021**
	Basal	618	0.79 (0.62–1.02)	0.075
Lymph node	Positive	1,133	1.74 (1.42–2.12)	**3.9e-08**
	Negative	2,020	1.7 (1.43–2.01)	**9.1e-10**
Grade	I	345	3.31 (1.81–6.04)	**3.7e-05**
	II	901	1.55 (1.22–1.98)	**0.00034**
	III	903	1 (0.8–1.24)	0.97
p53	Mutated	188	0.68 (0.42–1.09)	0.11
	Wild type	273	1.66 (1.08–2.54)	**0.019**

We also analyzed the impact of *FBXW7* and *Fascin* on patient survival, as regulation of these genes has been linked to SETD3^22^. *FBXW7* expression correlated with improved survival in ER-positive and Luminal A tumors (HR = 0.85; *P* = 0.012; HR = 0.78; *P* = 0.0046, respectively) and with worse survival in the Her-2 positive group (HR = 1.6; *P* = 0.034) (Table S4). High *Fascin* levels correlated with poor survival in Her-2 negative and Grade III tumors with a HR = 1.37; *P* = 0.018; HR = 1.34; *P* = 0.0091, respectively (Table S5). The other analyzed groups had no association with RFS.

Our investigation suggests that high tumoral levels of SETD3 are associated with a better outcome of breast cancer patients especially in ER-positive tumors. Moreover, in PR-negative and in triple-negative tumors a high expression of SETD3 results in reduced survival. Interestingly, in the presence of p53 mutations, the higher expression of SETD3 correlates with worse relapse-free survival even in ER-positive tumors. In addition, the HR of PR-negative tumors is worse when p53 is mutated and shows a high expression of SETD3. Finally, we observed that SETD3 regulates the invasive potential and the cytoskeleton of highly aggressive mammary cancer cells.

## Materials and Methods

### Kaplan-Meier plots and TCGA expression data

To assess the prognostic value of SETD3 and further functionally linked genes, the online tool kmplot.com^24^, which allows a meta-analysis of gene expression in relation to breast cancer patient survival, was employed. Gene expression data was obtained through microarray analysis of widely used arrays of the GEO database and converted into Kaplan-Meier plots^34^. This study includes relapse-free survival (n = 3,951) and overall survival (n = 1,402). The package “survival” was used in the R programming environment to plot Kaplan–Meier survival curves and compute the number-at-risk^24^. To distinguish between high and low expression, the median was selected as cut off-value to reduce the impact of outliers and produce equal numbers in both categories to only show strong correlations. Also, the *JetSet* probe set was selected to acquire unambiguous expression estimates^35^ and redundant samples were removed to enhance the quality of the sample. Patients were stratified by ER status, HER2 status, lymph node status, p53 status, molecular classification, Pietenpol subtype, and grading. We analyzed the expression of SETD3, FOXM1, FBXW7, and Fascin, and visualized the correlation to survival by drawing Kaplan-Meier survival plots. The Affymetrix IDs are 212465_at (SETD3), 202580_x_at (FOXM1), 218751_s_at (FBXW7), and 201564_s_at (FSCN1). The shown hazard ratios are not inverted (HR < 1 favorable). Gene expression data as plotted in Fig. 1 were derived from *The Pathology Atlas* section^34^ of the *Human Protein Atlas* (www.proteinatlas.org/)^35^, using gene expression data of *The Cancer Genome Atlas* (TCGA, https://www.cancer.gov/tcga). RNA-seq data in 17 cancer types were reported as the median number of fragments per kilobase of exon per million reads. RNA cancer tissue category is calculated based on mRNA expression levels across all cancer tissues and included cancer tissue enriched, cancer group enriched, cancer tissue enhanced, expressed in all, mixed and not detected. Normal distribution across the dataset was visualized with box plots, shown as median and 25th and 75th percentiles.

### Cell culture

All human breast cancer cell lines were purchased from ATCC/LGC Promochem (Wesel, Germany). MDA-MB-453, T47D, MDA-MB-468, MDA-MB-231, and SK-BR-3 cells were cultured in DMEM with 1% glutamine (cat. No. D0819) containing 10% fetal calf serum (FCS) (Biochrom GmbH, Cat. No. S0615, Berlin, Germany), and 1% penicillin/streptomycin (cat. No. P433) and maintained in a humidified atmosphere of 7.5% CO_2_ at 37 °C. MCF-7 cells were cultured in RPMI (Sigma, cat. No. D8758) containing 10% FCS, 1% glutamine and 1% penicillin/streptomycin maintained in a humidified atmosphere of 5% CO_2_ at 37 °C. BT474 cells were  cultured in RPMI containing 20% FCS, 1% glutamine and 1% penicillin/streptomycin and 0.01 mg/ml insulin (cat. No. 105) in a humidified atmosphere of 5% CO_2_ at 37 °C. All reagents were from Sigma-Aldrich Chemie GmbH, Taufkirchen, Germany, except for FCS.

### siRNA transfection

2.5 × 10^5^ cells/well of a six-well plate were cultured in DMEM medium for 24 hours. siRNA transfection was performed on semi-confluent cells (60%-70%) using Dharmafect reagent (Dharmacon™, cat. no. T-2001-03, Lafayette, CO, USA) according to the supplier’s instructions. This reagent contained in a total volume of 1 mL 840 µL Opti-MEM^®^ media (Gibco®, cat. no. 31985-070, Thermo-scientific, Germany), 80 µL 20 nM SETD3 siRNA/Opti-MEM^®^ (Ambion® life technologies, cat. no. 4392420, ID S38639, Cambridgeshire, UK) or negative control siRNA (Ambion® cat. no. 4390844, Cambridgeshire, UK), and 80 µL 2.5% Dharmafect/Opti-MEM^®^ solution. Cells were left for incubation at 37 °C and 7.5% CO_2_ for 24 hours and then the medium was changed to DMEM medium with FCS. mRNA and protein extraction was performed 48 hours after the initial transfection.

### Quantitative real-time PCR

mRNA isolation was performed with InnuPREP RNA mini kit (Analytikjena, cat. no. 845-KS-2040250, Jena, Germany), then reverse transcribed into cDNA using the High-Capacity cDNA Reverse Transcription Kit (Applied Biosystems, cat. No. 4368814, Foster City, CA, USA) according to the supplier’s protocols. Quantitative real-time PCR was performed in duplicates for each target gene using RT^2^ SYBR Green qPCR Primer Assay (Qiagen, cat. No. 330500, MD, USA) and gene expression levels were measured in an ABI 7300 Real-time PCR detection system (Applied Biosystems, CA, USA). Transcriptional analysis was performed using the ∆∆CT method and samples were normalized to the expression of beta-actin (β-actin) and glyceraldehyde-3-phosphate dehydrogenase (GAPDH) as internal controls. Melting curve analysis was performed to confirm specific product amplification. Primer sequences were confirmed by NCBI BLAST analysis and are listed in the Supplementary File Table S6.

### Western blot

Total cell lysates were prepared with protein Sample Buffer Laemmli 2×. 15 µl of protein per lane were separated on 10% SDS gels, and electro-transferred to nitrocellulose membranes (Amersham, Pharmacia Biotech, Piscataway, NJ, USA, cat. No. 10600008). Following 1 hour of blocking with 5% (w/v) skimmed milk powder in 1X TBST buffer, detection was performed with rabbit polyclonal primary antibody against SETD3 (1:1000, Sigma-Aldrich, cat no. HPA003639), and goat anti-rabbit IgG, H & L Chain Specific Peroxidase Conjugate (Sigma-Aldrich, cat. No. 401353). Then, membranes were subjected to a chemiluminescence reaction and signal quantification with NIH ImageJ software, normalizing the values of SETD3 to tubulin (1:10000, Sigma cat no. T5168; as a loading control). For tubulin detection, membranes were stripped with 0.2 M glycine buffer (pH 2.5), washed, re-incubated with primary antibody for tubulin as a housekeeping protein and subjected to the procedure described above.

### MTT proliferation assay

SETD3 depleted cells or controls (5 × 10^3^) were plated in 96‐well plates with DMEM medium (without phenol red) (Gibco®, cat. No. 31053028, Germany) with FCS and incubated for 96 hours. After the incubation time, the medium was removed completely and cells were incubated with 20 µl/well of 3-(4,5-Dimethylthiazol-2-yl)-2,5-diphenyl-tetrazolium bromide (MTT) (cat. No. M2128-1G), at 5 mg/ml for 4 hours, at 37 °C. Subsequently, 100 µl of Stopping buffer, pH 4.7 composed of 10% (w/v) sodium dodecyl sulfate (SDS) (cat. No. 3599286) and 50% (v/v) N,N-Dimethyl formamide (cat. No. 605365) was added to stop the reaction and dissolve the formazan crystals. The absorbance was measured in a VersaMax® Microplate Reader (Molecular Devices, Sunnyvale, CA, USA) at a wavelength of 595 nm. The proliferation of the control cells was defined as 100%. Results were derived from three independent sets of triplicate experiments. MTT, SDS, and N,N-Dimethyl formamide were from Sigma-Aldrich Chemie GmbH, Taufkirchen, Germany.

### Invasion assay

MCF-7 and MDA-MB-231 depleted SETD3 cells and control were diluted to 50.000 cells/mL in DMEM Media. Then, 500 µL (corresponding to 25.000 cells) were transferred to Matrigel wells (Corning®, cat. no. 354230; Bedford, MA). This was followed by a 24-hour incubation period (37 °C, 7.5% CO_2_). After carefully removing DMEM media, the invasion was triggered by filling the Matrigel wells with 800 µL of DMEM medium with FCS. After 24 hours, the FCS-free media was replaced by DMEM medium with FCS to remove the invasion stimulus. Then, all media was removed, and the cells on top of the Matrigel were removed with “cotton-wool” sticks and washed in PBS for 1 minute. After removing the PBS, the cells were stained in 1% toluidine blue in BORAX (Sigma, cat. No. T3260) for 6 minutes and washed with H_2_O. Two non-overlapping pictures were taken under a Zeiss Axiophot (Zeiss, Jena, Germany) bright-field microscope (magnification 10×) and invaded cells were counted.

### Collagen contraction assay

SETD3- and control siRNA-transfected MDA-MB-231 cells were harvested and re-suspended in DMEM media at 3 × 10^6^ cells/ml. Cell-embedded collagen gels were prepared on ice by neutralizing and diluting Type-I collagen solution (4.88 mg/ml; Corning, cat. No. 8274002, NY, USA) with NaOH, nuclease-free water, and 10X PBS into 2.5 mg/ml then cell suspension was immediately mixed at 2:8 ratio to collagen solution. The cell collagen mixture (500 µL/well) was immediately pipetted in a 24-well plate and placed in a culture incubator to polymerize at 37 °C for 60 minutes, then cell-seeded collagen gels were carefully detached from the well to remain free-floating in the culture medium. Subsequently, 1 mL of DMEM media with FCS was added to each well and incubated at 37 °C, 7.5% CO_2_ humidified atmosphere. The medium was changed every two days up to the decided time points. The collagen gel contraction progress was monitored by measuring the surface area of the gels every day^36^. The 24-well plate and cell-embedded gel were photographed after 24 hours and 72 hours using a digital camera with a resolution of 2160 × 1080 pixels and an Axiovert 100 microscope (Carl Zeiss, Jena, Germany). The digital images of the surface were analyzed and calculated using the NIH ImageJ program (U.S. National Institutes of Health, Bethesda, Maryland, USA). The contracted area was presented as a percentage of the initial surface area.

### Fluorescence microscopy

MDA-MB-231 cells (5 × 10^4^) were cultured on fibronectin-coated coverslips in 24-well plates after being transfected with negative control siRNA and SETD3 siRNA transfection for 24 hours. The medium was removed completely, then cells were fixed with 3.7% paraformaldehyde (Merck KGaA, K42464803, Darmstadt, Germany) for 10 minutes, followed by a 1-minute incubation in 0.1% Triton X-100 (Carl Roth GmbH and Co. KG, 3051.3, Austria). Cells were washed twice with 1 × PBS for 5 minutes each and incubated for 30 minutes with Aurion BSA 10% (AURION, 60613/3, Wageningen, Netherlands) (1:10). For staining of actin filaments, cells were incubated for 30-minutes with Phalloidin CruzFluor™ 594 Conjugate 1000 × (1:500, Santa Cruz Biotechnology, sc-363795, Texas, USA) in Dako REAL^TM^ Antibody Diluent (Agilent Technologies, S2022, California, USA). Subsequently, samples were washed for three times with PBS for 5 minutes each and then incubated with 4′,6′-diamidino-2-phenylindole (DAPI, dilactate) (Sigma-Aldrich Chemie GmbH, D9564, Taufkirchen, Germany) (1:5000 in PBS) for an additional 1 minute, followed by rinsing with PBS. Cell-containing coverslips were applied to a drop of Dako Fluorescence Mounting Medium (Agilent Technologies, S3023, California, USA) on glass slides. Samples were examined by confocal immunofluorescence microscopy on a Zeiss LSM 780 (Carl Zeiss, Jena, Germany) using Plan-Apochromat 40x Oil DIC objectives.

### Protein interaction network analysis

STRING v11 (http://string-db.org/)^33^ was used to generate *in silico* protein interaction networks for the gene products that we analyzed in KMplot, SETD3, FOXM1, FBXW7, ACTB, FSCN1, MMP-2, KLC4, iNOS, and eNOS. All interactions are predicted with a high confidence threshold of 0.700, and all active predictive methods were allowed. For the enrichment analysis, STRING implements well-known classification systems such as Gene Ontology (GO) and KEGG (Kyoto Encyclopedia of Genes and Genomes)^33^.

### Statistical analyses

For survival analysis, in the R statistical environment, we utilized the Kaplan-Meier-Plotter database via the statistical package “survival” to calculate Kaplan–Meier survival curves and the number-at-risk. Furthermore, the hazard ratio (and 95% confidence intervals) and log-rank P were calculated for each gene^24^. For experimental assays, statistical analysis was performed with GraphPad Prism 4.02 (GraphPad Software, La Jolla, CA). GraphPad Prism 4.02 was used to perform two-tailed t-tests, one-way ANOVA with Dunn´s posttest, or nonparametric Friedman’s test with Dunn’s posttest, where appropriate. Significant P-values are indicated as follows: ≤0.05 by one asterisk *, ≤0.01 by two asterisks ** and ≤0.001 by three asterisks ***.

## Discussion

One of the most studied epigenetic modifications is the methylation of DNA. Under physiological conditions, it contributes to the regulation of the expression of genes that must be expressed in a defined physiological state, while silencing those that should not^37^. Since DNA modification is a pivotal player in transcriptional regulation, defects in this mechanism may lead to tumorigenesis and cancer progression^38^. Already in pre-invasive stages of cancer, hypermethylation leads to the abnormal silencing of some genes such as p16 and glutathione S-transferase-π1 (GSTP1), allowing neoplastic cells to progress towards more aggressive stages^39^. Notably, the methylation of actin plays an important role in the remodeling of the cytoskeleton which occurs during development and steady-state physiology, and in processes associated with cancer like invasion and metastasis, promoted by epithelial to mesenchymal transition (EMT)^17,40,41^. Also, the methylation of actin on His73 by SETD3, a novel methyltransferase, prevents primary dystocia^10^. Interestingly, the human methyltransferasome includes 208 known members, of which 22 SETD proteins have been associated with different diseases including cancer in both humans and animal models^42,43^. Previously, the expression of SETD3 has been associated with oncogenesis of lymphoma and liver cancer^44^. In breast cancer, however, our study indicates a bivalent role of SETD3.

In the present study, we found that the expression of SETD3 was associated with better relapse-free survival in the patients in general without any classification, and in patients with ER-positive and Luminal A-type tumors. In contrast, in PR-negative and triple-negative tumors (ER-, PR- and HER2-negative), SETD3 is associated with poor RFS. Most importantly, in breast cancer patients with mutated p53, the expression of SETD3 is linked to worse prognosis even in patients with ER-positive tumors. Although it has been demonstrated that SETD3 is required for p53 target gene recruitment and the induction of apoptosis in response to DNA damage in cancer colon cells^23^, in our study we observed that downregulation of SETD3 is associated with reduced viability of aggressive breast cancer cells. In agreement with that, in hepatocellular carcinoma cells, the knockdown of SETD3 markedly reduced cell proliferation^22^ and in consequence resulted in the formation of small tumors *in* *vivo*. Interestingly, in the same study, the expression of SETD3 was higher in specimens of liver cancer patients with grade III^22^. We found this information important because in breast cancer as well as other types of cancer the grade is associated with metastasis to lymph nodes^45^. However, while we obtained a high HR score regarding the expression of SETD3 and grade 3, we found no significant difference (Table 1). It seems that SETD3 has different roles in cancer depending on other mutations and the stage of tumor progression^22,23,32^. This is not surprising since it has been reported that other molecules have dual roles and can act as anti-tumoral and/or pro-tumoral factors. For example, the bone morphogenetic proteins (BMPs) that are part of the transforming growth factor-β (TGF-β) superfamily have been related to the induction of angiogenesis, EMT, cancer stem cells (CSCs) and metastasis in aggressive breast, skin, and prostate cancer cells lines and tissues^46^. On the other hand, BMP10 which is part of the BMP family suppresses the aggressiveness of prostate cancer cells by the induction of apoptosis^47^ and in patients with low expression of BMPRIB exhibited poor prognosis, especially in the luminal subtype^48^. Also, the same group reported that patients that were treated with taxane and anthracycline regimens and have high expression of BMPRIB have a favorable prognosis^48^. Another clear example is TGF-β, which has a tumor-suppressive effect in non-tumorous cells and pre-malignant stages, while it facilitates tumor metastasis in late-stage cancer cells^49^.

An important result of our *in vitro* analysis is that SETD3 siRNA treated MDA-MB-231 cells, representing the basal subtype, were 35% less invasive than controls, whereas no significant impact on invasiveness was noted for SETD3 knockdown in the luminal cell line MCF-7 as the MDA-MB-231 cell line displays a highly invasive phenotype^50^, this could be explained by the already described effects of SETD3 on actin polymerization and cell metabolism^10^. Apparently the cytoskeleton of the highly metastatic basal MDA-MB-231 cells was affected in a stronger manner as the less dedifferentiated luminal cell line MCF-7. The cell-type-specific alterations in cytoskeletal gene expression (Fascin, ACTG2, ASMA) may be indicative of a differential effect on the cytoskeleton (Fig. 6). Indeed, numerous studies have demonstrated that the actin cytoskeleton of basal and luminal breast cancer cells shows structural and functional differences that result e.g. in an increased actin response in the interaction of breast cancer cells with natural killer cells^51^, in differential nuclear shape dynamics^52^, in the suppression of invasion^53^, and in cell fusion and the formation of cancer hybrid cells^54^. Other SETD proteins such as SETD5 have been associated with the invasion and migration of non-small cell lung cancer cells^55^. Interestingly, the authors found that SETD5 regulates the expression of master EMT transcription factors like Snail and ZO-1^55,56^. They also found that higher SETD5 expression was significantly correlated with advanced TNM stage, lymph node metastasis, and overall survival rate. The invasion process is characterized by a reorganization of the actin cytoskeleton, and the formation of membrane protrusions is a requirement for invasive growth^57^. Our morphological analysis indicates that MDA-MB-231 SETD3 depleted cells undergo changes in their cytoskeleton arrangement and lose their large membrane protrusions. This might be the result of a higher frequency of actin polymerization and destabilization of the actin cytoskeleton through hypomethylation^10^ and could explain their less invasive capacity. Our finding of a substantially reduced capability of SETD3-depleted MDA-MB-231 cells to contract collagen gels supports this observation at the functional level, as it is indicative of impaired cytoskeletal function.

According to our and other studies, it seems that SETD3 significantly regulates mechanisms associated with actin regulation, however, the protein network in which SETD3 is involved is unknown. Cohn *et al*. demonstrated that SETD3 can interact with 172 novel proteins such as transcription factors of which FoxM1 was one of them^21^. Also, SETD3 methylates FoxM1 and negatively regulates the expression of Vascular Endothelial Growth Factor (VEGF) under normoxia conditions^21^, a known factor related to angiogenesis and tumor progression^58^. On the other hand, FoxM1 has an important role in cancer because its overexpression has been found to be related to poor prognosis in patients^29^. In our study, we found that the expression of FoxM1 also correlated with worse RFS in breast cancer patients (Table 2). When we analyzed the expression of both genes at the same time in the patients classified as ER-positive and Luminal A, we observed worse survival. However, the real-time gene expression analysis revealed that SETD3 knockdown influences the expression of FoxM1 which was downregulated (Fig. 6). Furthermore, the STRING analysis showed that SETD3 has a very close interaction with FoxM1 (Fig. 7). Our study could, therefore, confirm the observation of Cohn *et al*. that SETD3 regulates the expression of FoxM1^21^. Nevertheless, the discrepancy that we observed in our Kaplan-Meier analysis where the SETD3 expression correlates with a good prognosis while FOXM1 with a bad prognosis in patients with ER-positive tumors could be due to the following reasons: 1) the expression of FOXM1 (up- or down-regulation) is not only regulated by SETD3 but is also regulated by a wide range of factors such as c-Myc, H-Ras, N-Ras, EGFR (epidermal growth factor receptor), ErbB1, HER2/Neu (ErbB2), Notch1, STAT3 (signal transducer and activator of transcription 3), KLF4, GATA3, and others^59^. 2) Interestingly, it has been described that ERα activates FOXM1 transcription through an estrogen-response element (ERE) located within the proximal promoter region^59,60^. This could explain why even in ER-positive tumors the expression of FOMX1 correlates with a poor prognosis. On the other hand, Carr and collaborators observed that the expression of FoxM1 is highest in luminal progenitor cells and decreases upon differentiation. Thus, FOXM1 regulates the differentiation of the luminal cells in the breast and that later could lead to more aggressive characteristics in neoplastic cells^61^. 3) The expression of FOXM1 was associated with processes related to aggressiveness like EMT, CSCs, drug resistance and is a master regulator of metastasis, which is the ultimate cause of patient mortality^59,62–64^. All of the above could explain why although FOXM1 is regulated by SETD3 (which is associated with a good prognosis), it was associated with worse survival in patients.

Another gene that could be regulated by SETD3 is Fascin. We observed the downregulation of Fascin in SETD3 depleted-cells that suggest a negative regulatory mechanism of Fascin directly through the expression of SETD3. In a meta-analysis study, Fascin expression was associated with an increased risk of mortality in the breast, colorectal and gastric cancers^65,66^. Interestingly, Heinz and colleagues demonstrated that the expression of Fascin was up-regulated in highly aggressive MDA-MB-231 breast cancer cells and metastasis was also increased by an actin bundling-independent mechanism^67^. Another study observed that the downregulation of Fascin alters cell morphology and reduces cell migration and invasion, while the overexpression of this gene and increases the expression of genes associated with metastasis such as matrix metalloproteases (MMP)-2 and -9 and urokinase-type plasminogen activator (uPA)^68^. Finally, microRNA-mediated targeting of Fascin is part of the anti-invasive mechanism of the tumor suppressor miR-145 in breast cancer^69^. In contrast to our observation that Fascin only has clinical implications in Her2-negative and grade 3 tumors (Table S5), another study revealed that Fascin expression is associated with poor survival in ER-negative tumors^70^. Methodological differences may account for this discrepancy, as the aforementioned study analyzed tissue microarrays, whereas our study relied on transcriptomic analyses. Moreover, the patient collective of our study was 19-fold larger than the collective of Yoder *et al*.^70^.

Our qPCR analysis revealed that SETD3 siRNA treatment in luminal MCF-7 cells was associated with an upregulation of three pathogenesis factors that were not significantly affected in basal MDA-MB-231 cells, ASMA, MMP2, and eNOS. ASMA is an actin isoform that is upregulated in the tumor and promotes in cancer cells a metastatic activity associated to EMT^71,72^, MMP2 is a metalloproteinase that promotes metastatic growth by degrading basement membrane constituents^73,74^, whereas eNOS affects inflammation, angiogenesis, apoptosis, cell cycle, invasion, and metastasis^75,76^. Therefore, conversely, high expression of SETD3 in ER-positive tumors may result in a survival benefit due to a downregulation of these factors that is worth addressing in future studies.

In summary, our results suggest that the expression of SETD3 influences the survival of breast cancer patients. Particularly in triple-negative and p53 mutated tumors, SETD3 emerges as an important target for the treatment of BC patients. On the other hand, SETD3 expression was related to good prognosis in ER-positive tumors which suggests that its prognostic value and pathophysiological role depend on the expression of hormone receptors and p53. Additionally, elucidating its role in invasion and metastasis as well as in the regulation of the expression of genes such as FOXM1, MMP2, eNOS, ASMA, and Fascin could point out new therapeutic strategies for more specific treatments. Importantly, our results indicate SETD3 among the new potential targets in TP53 tumors as to date no targeted therapy is available for this important sub-cohort of patients. While a differential effect on the actin cytoskeleton and invasiveness of basal and luminal model cell lines have emerged as an underlying mechanism linked to metastatic behavior, the study of additional mechanisms by which SETD3 acts as a marker of good prognosis in receptor-positive tumors and as a marker for poor prognosis in triple-negative tumors will help us to better understand the role of SETD3 in tumor progression.

## Supplementary information

Supplementary information

## Data Availability

All data generated or analyzed during this study are included in this published article and its Supplementary Information Files.
