# Supplementary material for: SETD3 acts as a prognostic marker in breast cancer patients and modulates the viability and invasion of breast cancer cells
Source: Sci Rep. 2020 Feb 10;10:2262. doi: 10.1038/s41598-020-59057-5 (PMC7010743; doi:10.1038/s41598-020-59057-5)
Supplement: Supplementary file 1 — Supplementary information [file 41598_2020_59057_MOESM1_ESM.pdf]

Online supplementary material for

SETD3 acts as a prognostic marker in breast cancer patients and modulates the viability and invasion of breast cancer cells

Hassan, Nourhan[1+]; Rutsch, Niklas[1+]; Győrffy, Balázs[2]; Espinoza-Sanchez, Nancy Adriana[3§]; Götte, Martin[1§*]

[1]Department of Gynecology and Obstetrics, Münster University Hospital, Albert-Schweitzer-Campus 1, D11, Münster, 48149, Germany

[2]MTA TTK Lendület Cancer Biomarker Research Group, Institute of Enzymology, Hungarian Academy of Sciences, and Semmelweis University 2nd Dept. of Pediatrics, Budapest, Hungary.

[3]Unidad de Investigación en Virología y Cáncer, Hospital Infantil de México Federico Gómez, Ciudad de México, 06720, Mexico.

This document contains: Supplementary tables and supplementary figures

Table S1. GO enrichment analysis associated with the SETD3, FOXM1, FBXW7, ACTB, MMP-2, KLC4, iNOS, and eNOS. The program STRING was used for this analysis.

Category	Pathway description	Observed gene count	False discovery rate	Matching proteins in your network (labels)
Molecular function	Actin binding	6	0.0001	CFL1,FSCN1,FSCN2,FSCN3,NOS3,PFN1
	Cytoskeletal protein binding	7	0.00026	ACTB,CFL1,FSCN1,FSCN2,FSCN3,NOS3,PFN1
	Nitric-oxide synthase activity	2	0.00035	NOS2,NOS3
	Tetrahydrobiopterin binding	2	0.00035	NOS2,NOS3
	Actin filament binding	4	0.00035	CFL1,FSCN1,FSCN2,FSCN3
	NADPH-hemoprotein reductase activity	2	0.00037	NOS2,NOS3
	Protein binding	14	0.00037	ACTB,AKT1,CFL1,FBXW7,FOXM1,FSCN1,FSCN2,FSCN3,NOS2, NOS3,PFN1,SETD3,SKP1,TIMP2
	Protein binding, bridging	4	0.00037	FBXW7,FSCN1,FSCN2,FSCN3
	Arginine binding	2	0.00038	NOS2,NOS3
	FMN binding	2	0.001	NOS2,NOS3
Cellular component	Cytoskeleton	10	5.66E-05	ACTB,AKT1,CFL1,FSCN1,FSCN2,FSCN3,KLC4,NOS2,NOS3,PFN 1
	Filamentous actin	3	5.66E-05	FSCN1,FSCN2,FSCN3
	Intracellular non-membrane-bounded organelle	13	5.66E-05	ACTB,AKT1,CFL1,FBXW7,FSCN1,FSCN2,FSCN3,KLC4,MMP2,N OS2,NOS3,PFN1,SETD3
	Actin cytoskeleton	5	0.00065	ACTB,CFL1,FSCN1,FSCN2,FSCN3
	Organelle part	15	0.0023	ACTB,AKT1,CFL1,FBXW7,FOXM1,FSCN1,FSCN2,FSCN3,KLC4, MMP2,NOS2,NOS3,SETD3,SKP1,TIMP2
	ruffle	3	0.0061	CFL1,FSCN1,FSCN3
	Growth cone	3	0.0061	FSCN1,FSCN3,TIMP2
	Lamellipodium	3	0.0068	CFL1,FSCN1,FSCN3
	Cell cortex	3	0.007	CFL1,NOS2,PFN1
	SCF ubiquitin ligase complex	2	0.007	FBXW7,SKP1
Biological process	Nitric oxide biosynthetic process	3	0.00037	AKT1,NOS2,NOS3
	Organelle organization	12	0.00037	ACTB,AKT1,CFL1,FBXW7,FSCN1,FSCN2,FSCN3,NOS2,NOS3,PF N1,SETD3,SKP1

	Cellular component organization	14	0.00037	ACTB,AKT1,CFL1,FBXW7,FSCN1,FSCN2,FSCN3,MMP2,NOS2,NOS3,PFN1,SETD3,SKP1,TIMP2
	Actin cytoskeleton organization	6	0.00037	ACTB,CFL1,FSCN1,FSCN2,FSCN3,PFN1
	Positive regulation of cyclase activity	3	0.00037	NOS2,NOS3,TIMP2
	Nitric oxide metabolic process	3	0.00037	AKT1,NOS2,NOS3
	Positive regulation of lyase activity	3	0.00037	NOS2,NOS3,TIMP2
	Establishment or maintenance of cell polarity	4	0.00097	CFL1,FSCN1,FSCN2,FSCN3
	Cytokine-mediated signaling pathway	6	0.00097	AKT1,CFL1,FSCN1,MMP2,NOS2,SKP1
	Response to cytokine	7	0.00097	AKT1,CFL1,FSCN1,MMP2,NOS2,SKP1,TIMP2

Table S2. Biological process associated with SETD3, FOXM1, FBXW7, ACTB, MMP-2, KLC4, iNOS, and eNOS according to KEEG enrichment analysis. The program STRING was used for this analysis.

Pathway description	Observed gene count	False discovery rate	Matching proteins in your network (labels)
Salmonella infection	4	8.05E-05	ACTB,KLC4,NOS2,PFN1
Relaxin signaling pathway	4	0.00022	AKT1,MMP2,NOS2,NOS3
Fluid shear stress and atherosclerosis	4	0.00022	ACTB,AKT1,MMP2,NOS3
HIF-1 signaling pathway	3	0.0021	AKT1,NOS2,NOS3
AGE-RAGE signaling pathway in diabetic complications	3	0.0021	AKT1,MMP2,NOS3
Arginine biosynthesis	2	0.0028	NOS2,NOS3
Apelin signaling pathway	3	0.0028	AKT1,NOS2,NOS3
Platelet activation	3	0.0028	ACTB,AKT1,NOS3
Estrogen signaling pathway	3	0.0028	AKT1,MMP2,NOS3
Proteoglycans in cancer	3	0.0063	ACTB,AKT1,MMP2
Rap1 signaling pathway	3	0.0064	ACTB,AKT1,PFN1
Regulation of actin cytoskeleton	3	0.0064	ACTB,CFL1,PFN1
Pathways in cancer	4	0.0064	AKT1,MMP2,NOS2,SKP1
Arginine and proline metabolism	2	0.0065	NOS2,NOS3
VEGF signaling pathway	2	0.009	AKT1,NOS3
Shigellosis	2	0.0096	ACTB,PFN1
Pertussis	2	0.0123	CFL1,NOS2
Fc gamma R-mediated phagocytosis	2	0.0165	AKT1,CFL1
Small cell lung cancer	2	0.0167	AKT1,NOS2
Endocrine resistance	2	0.0169	AKT1,MMP2
Chagas disease (American trypanosomiasis)	2	0.0181	AKT1,NOS2

Table S3. Statistical co-citation analysis associated with SETD3, FOXM1, FBXW7, ACTB, MMP-2, KLC4, iNOS, and eNOS according to PubMed. The program STRING was used for this analysis.

Pathway description	Observed gene count	False discovery rate	Matching proteins in your network (labels)
(2014) Extracellular vesicles as emerging intercellular communicasomes.	6	2.42E-06	ACTB,AKT1,CFL1,MMP2,PFN1,TIMP2
(2007) Nanostructured HA crystals up-regulate FGF-2 expression and activity in microvascular endothelium promoting angiogenesis.	4	4.73E-06	ACTB,AKT1,MMP2,NOS3
(2017) MicroRNA as Therapeutic Targets for Chronic Wound Healing.	5	4.73E-06	AKT1,CFL1,MMP2,NOS2,NOS3
(2011) The relationship between endotoxemia and hepatic endocannabinoids in cirrhotic rats with portal hypertension.	4	5.41E-06	AKT1,MMP2,NOS3,TIMP2
(2005) Effects of rapamycin on the arterial inflammatory response in atherosclerotic plaques in Apo-E knockout mice.	4	6.49E-06	AKT1,MMP2,NOS3,TIMP2
(2016) Effects of Nitrate Intake on Myocardial Ischemia-Reperfusion Injury in Diabetic Rats.	4	6.49E-06	ACTB,AKT1,NOS2,NOS3
(2014) Female resistance to pneumonia identifies lung macrophage nitric oxide synthase-3 as a therapeutic target.	4	6.70E-06	ACTB,AKT1,NOS2,NOS3
(2008) Oxidative and nitrosative stress and fibrogenic response.	5	8.20E-06	AKT1,MMP2,NOS2,NOS3,TIMP2
(2010) Flavonoids: antioxidants against atherosclerosis.	4	8.20E-06	AKT1,MMP2,NOS2,NOS3
(2012) Acute and chronic cardioprotection by the enkephalin analogue, Eribis peptide 94, is mediated via activation of nitric oxide synthase and adenosine triphosphate-regulated potassium channels.	4	8.20E-06	AKT1,MMP2,NOS2,NOS3

Table S4. Correlation between the expression of FBXW7 and the status of breast cancer patients according different classifications.

**Classification**	**status**	**Cases**	**HR 95% CI**	***P* value**
ALL	-	3,951	0.9 (0.81 – 1.01)	0.067
Estrogen receptor	Positive	3,082	0.85 (0.75 – 0.97)	**0.012**
	Negative	869	0.98 (0.79 – 1.21)	0.85
Progesterone receptor	Positive	589	1.05 (0.74 – 1.49)	0.77
	Negative	549	1.14 (0.85 – 1.52)	0.39
Her2	Positive	252	1.6 (1.03 – 2.48)	**0.034**
	Negative	800	0.82 (0.63 – 1.06)	0.13
Intrinsic subtype	Luminal A	1,933	0.78 (0.66 – 0.93)	**0.0046**
	Luminal B	1,149	0.94 (0.78 – 1.14)	0.56
	Her2	251	1.01 (0.69 – 1.47)	0.98
	Basal	618	0.97 (0.75 – 1.25	0.81
Lymph node	Positive	1,133	0.93 (0.77 – 1.13)	0.48
	Negative	2,020	1.03 (0.87 – 1.22)	0.69
Grade	I	345	1.34 (0.8 – 2.25)	0.27
	II	901	1.12 (0.88 – 1.43)	0.35
	III	903	1.01 (0.82 – 1.26)	0.9
p53	Mutated	188	1.02 (0.64 – 1.64)	0.93
	Wild type	273	0.92 (0.6 – 1.4)	0.69

Table S5. Correlation between the expression of FSCN1 (Fascin-1) and the status of breast cancer patients according different classifications.

Classification	status	Cases	HR 95% CI	*P* value
ALL	-	3,951	1.1 (0.99 – 1.23)	0.081
Estrogen receptor	Positive	3,082	0.99 (0.87 – 1.12)	0.84
	Negative	869	1.07 (0.87 – 1.32)	0.51
Progesterone receptor	Positive	589	1.39 (0.97 – 1.98)	0.068
	Negative	549	1.26 (0.94 – 1.69)	0.12
Her2	Positive	252	1 (0.65 – 1.54)	1
	Negative	800	1.37 (1.05 – 1.78)	**0.018**
Intrinsic subtype	Luminal A	1,933	0.89 (0.75 – 1.05)	0.16
	Luminal B	1,149	1.14 (0.94 – 1.38)	0.18
	Her2	251	0.78 (0.53 – 1.14)	0.2
	Basal	618	1.03 (0.8 – 1.32)	0.82
Lymph node	Positive	1,133	1.19 (0.97 – 1.44)	0.089
	Negative	2,020	1.07 (0.91 – 1.27)	0.4
Grade	I	345	1.33 (0.79 – 2.25)	0.28
	II	901	1 (0.79 – 1.27)	0.98
	III	903	1.34 (1.07 – 1.66)	**0.0091**
p53	Mutated	188	0.82 (0.51 – 1.31)	0.4
	Wild type	273	0.78 (0.51 – 1.19)	0.24

Table S6. Sequences of primer pairs

SEDT3	Forward: 5'-CAA CCT GGA AGA TGA CCG CTG T-3'<br>Reverse: 5'-CAC TGT GGA TCA CAA ACT CTG CG-3'
GAPDH	Forward: 5'-CCCAGCAAGAGCACAAGAGG-3'<br>Reverse: 5'-GGTCTACATGGCAACTGTGAGGA-3'
FoxM1	Forward: 5'-CGT CGG CCA CTG ATT CTC AAA-3'<br>Rev: 5'-GGC AGG GGA TCT CTT AGG TTC-3'
FBXW7-beta	Forward: 5'-AGT ACC ACT GGG CTT GTA CC-3'<br>Rev: 5'-CTC TGG TCC ACT CCA GCT CT-3'
ASMA	Forward: 5'-ACTGAGCGTGGCTATTCCTCCGTT-3'<br>Reverse: 5'-GCAGTGGCCATCTCATTTTCA-3'
Beta-Actin	Forward: 5'-CAA AGA CCT GTA CGC CAA CAC-3'<br>Reverse: 5'-CAT ACT CCT GCT TGC TGA TCC-3'
Actin gamma 2	Forward: 5'-ATG GAA GGA AAC ACG GCT C-3'<br>Reverse: 5'-CAC TCT GTT CTT CCG CCG-3'
Fascin	Forward: 5'-GGA GAC CGA CCA GGA GAC-3'<br>Reverse: 5'-CAT TGG ACG CCC TCA GTG-3'
eNOS	Forward: 5'-TGG ACC TGG ATA CCC GGA C-3'<br>Reverse: 5'-TGG TGA CTT TGG CTA GCT GGT-3'

iNOS	Forward: 5’-GTT CTC AAG GCA CAG GTC TC-3’<br>Reverse: 5’-GCA GGT CAC TTA TGT CAC TTA TC-3’
KLC4	Forward: 5’-CAA CAA TTT GGC TGT GCT CT-3’<br>Reverse: 5’-TTT GCC ACA TCT GGA TGA TT-3’
MMP2	Forward: 5’-AGC GAG TGG ATG CCG CCT TTA A-3’<br>Reverse: 5’-CAT TCC AGG CAT CTG CGA TGA G-3’

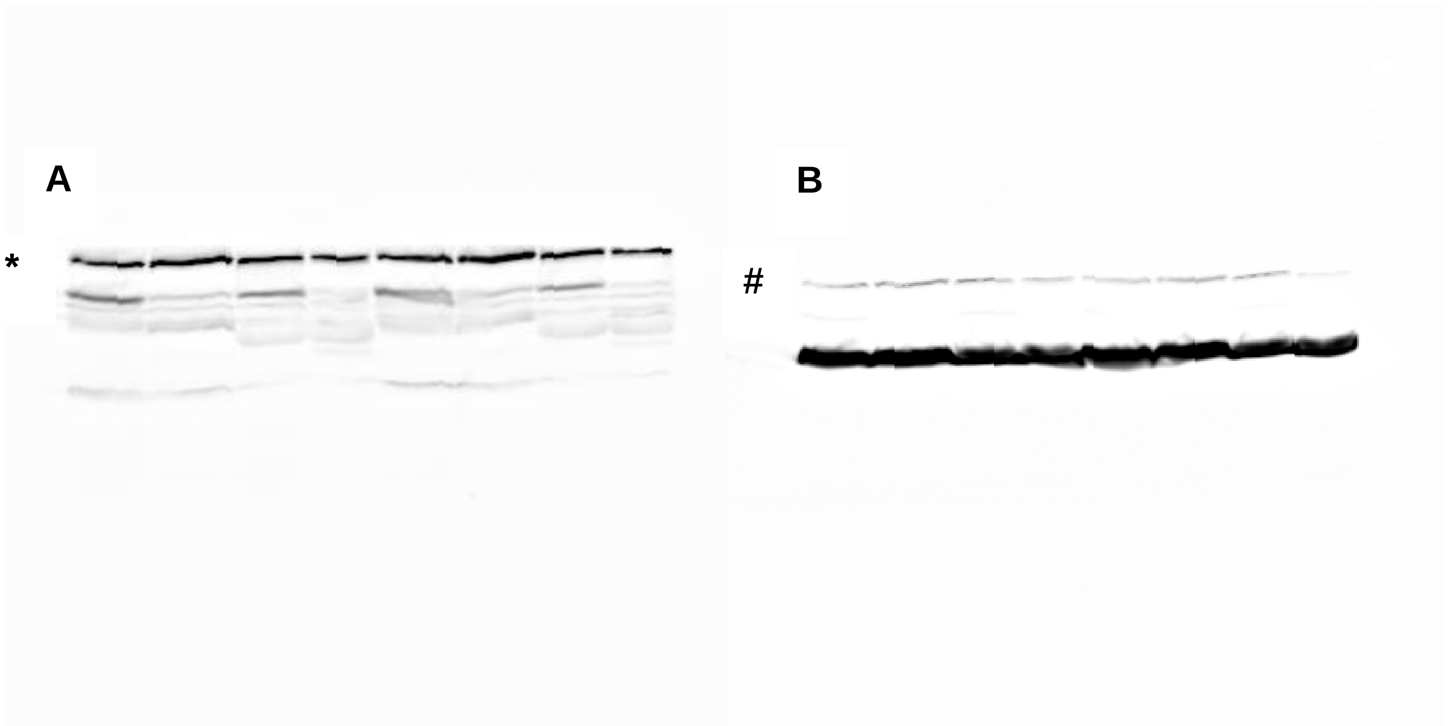
A
*
B
#

C

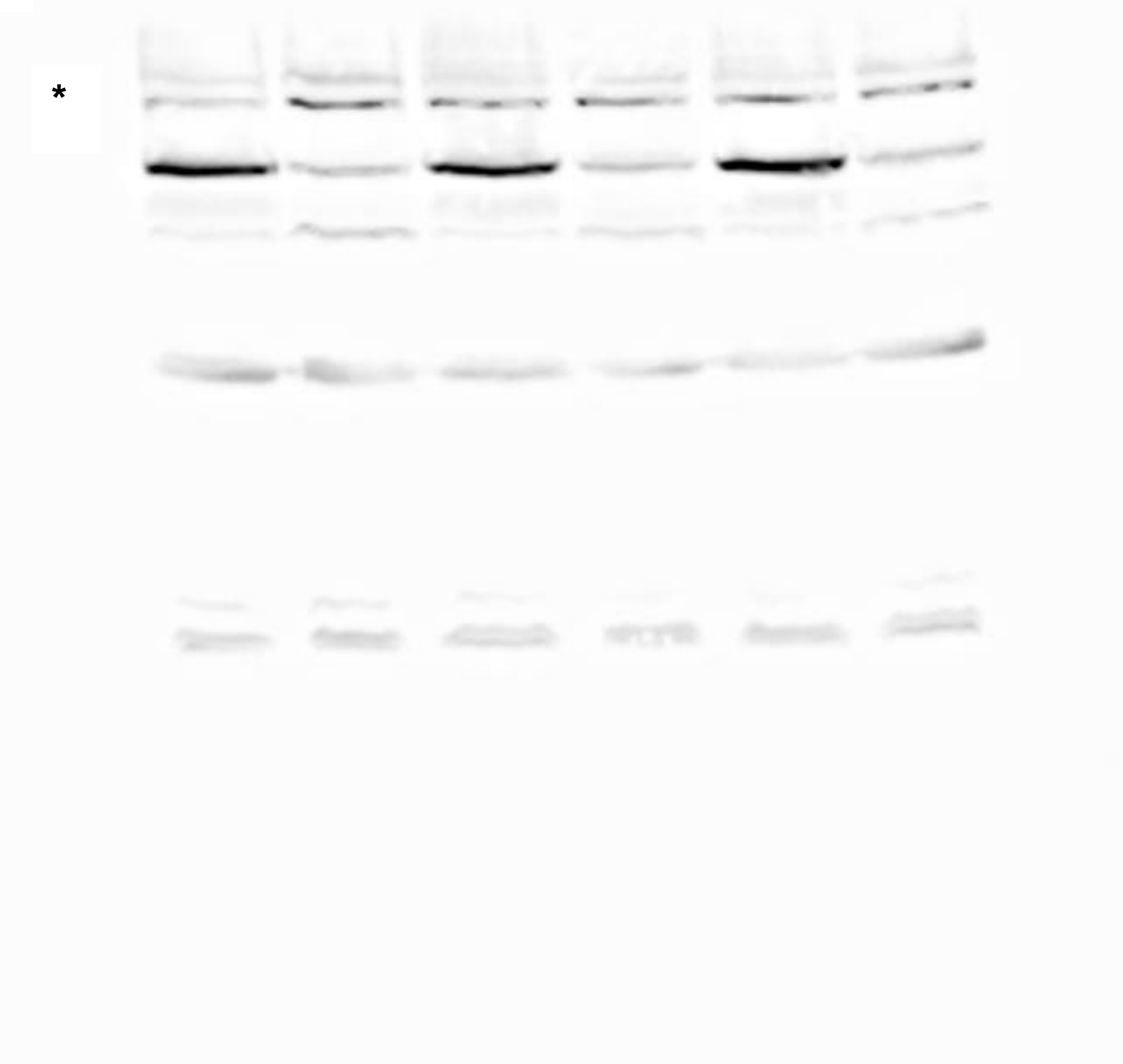

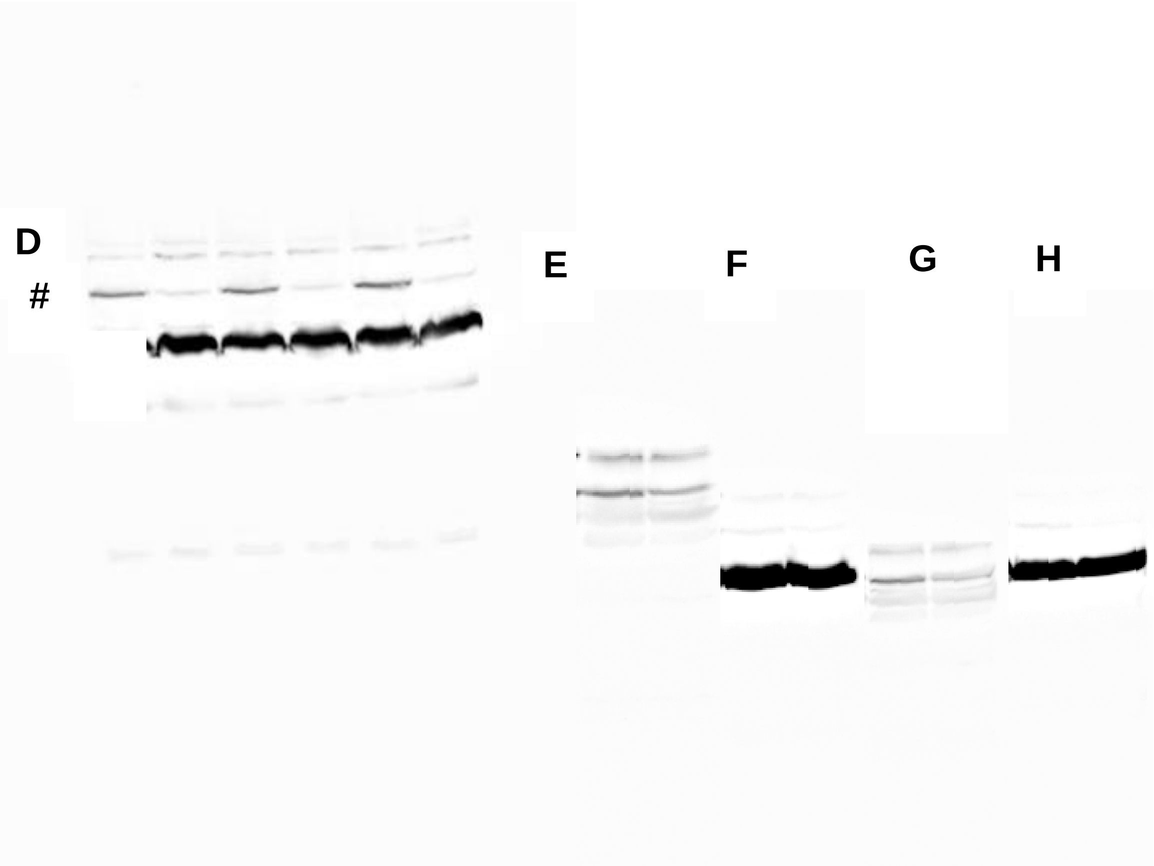

Supplementary Figure S1. Full-length original files of the Western blot signals shown in Figure 4C of the original manuscript. * indicates non-specific crossreactivity of the SETD3 antibody. # indicates non-completely stripped signal of SETD3 on tubulin-antibody reprobed stripped membranes. A) MDA-MB-231 and MDA-MB-468 SETD3 signal. B) MDA-MB-231 and MDA-MB-468 tubulin signal. C) MCF-7 SETD3 signal. D) MCF-7 tubulin signal. E) T47D SETD3 signal (left lanes Fig. 4C). F) T47D tubulin signal (left lanes Fig. 4C). G) T47D SETD3 signal (right lanes Fig. 4C). H) T47D tubulin signal (right lanes Fig. 4C).

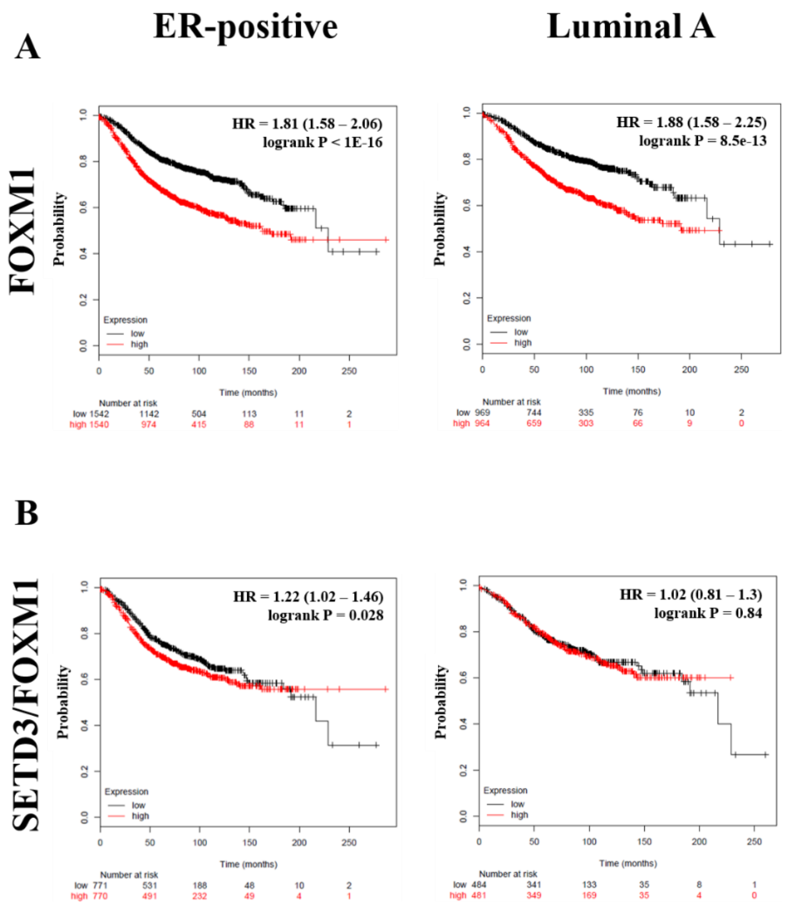

Supplementary Figure S2. The prognostic value of the expression of FOXM1 and the co-expression of SETD3/FOXM1 in patients with breast cancer stratified by hormone receptor status. Kaplan-Meier relapse-free survival curves are plotted based on: A) the expression of FOXM1 in patients with tumors Estrogen receptor (ER)-positive (left panel) and subtype Luminal A (right panel). B) the co-expression of SETD3/FOXM1 also in ER-positive and Luminal tumors. Log-rank p values and hazard ratios (HRs; 95 % confidence interval in parentheses) are shown. The corresponding Affymetrix IDs is: 212465_at_SETD3 and 202580_x_at FOXM1.
